# Impact of contraceptive initiation on vaginal microbiota

**DOI:** 10.1016/j.ajog.2018.02.017

**Published:** 2018-06

**Authors:** Sharon L. Achilles, Michele N. Austin, Leslie A. Meyn, Felix Mhlanga, Zvavahera M. Chirenje, Sharon L. Hillier

**Affiliations:** a Department of Obstetrics, Gynecology, and Reproductive Sciences and Center for Family Planning Research, University of Pittsburgh School of Medicine, Pittsburgh, PA; b Magee-Womens Research Institute, Pittsburgh, PA; c University of Zimbabwe-University of California, San Francisco Collaborative Research Program, Harare, Zimbabwe

**Keywords:** bacterial vaginosis, hormonal contraception, intrauterine device, lactobacilli, vaginal microbiota

## Abstract

**Background:**

Data evaluating the impact of contraceptives on the vaginal microbiome are limited and inconsistent.

**Objective:**

We hypothesized that women initiating copper intrauterine device use would have increased bacterial vaginosis and bacterial vaginosis-associated microbes with use compared to women initiating and using hormonal contraceptive methods.

**Study Design:**

Vaginal swabs (N = 1047 from 266 participants seeking contraception) for Nugent score determination of bacterial vaginosis and quantitative polymerase chain reaction analyses for assessment of specific microbiota were collected from asymptomatic, healthy women aged 18-35 years in Harare, Zimbabwe, who were confirmed to be free of nonstudy hormones by mass spectrometry at each visit. Contraception was initiated with an injectable (depot medroxyprogesterone acetate [n = 41], norethisterone enanthate [n = 44], or medroxyprogesterone acetate and ethinyl estradiol [n = 40]), implant (levonorgestrel [n = 45] or etonogestrel [n = 48]), or copper intrauterine device (n = 48) and repeat vaginal swabs were collected after 30, 90, and 180 days of continuous use. Self-reported condom use was similar across all arms at baseline. Quantitative polymerase chain reaction was used to detect *Lactobacillus crispatus*, *L jensenii*, *L gasseri*/*johnsonii* group, *L vaginalis*, *L iners*, *Gardnerella vaginalis*, *Atopobium vaginae*, and *Megasphaera*-like bacterium phylotype I from swabs. Modified Poisson regression and mixed effects linear models were used to compare marginal prevalence and mean difference in quantity (expressed as gene copies/swab) prior to and during contraceptive use.

**Results:**

Bacterial vaginosis prevalence increased in women initiating copper intrauterine devices from 27% at baseline, 35% at 30 days, 40% at 90 days, and 49% at 180 days (*P* = .005 compared to marginal prevalence at enrollment). Women initiating hormonal methods had no change in bacterial vaginosis prevalence over 180 days. The mean increase in Nugent score was 1.2 (95% confidence interval, 0.5–2.0; *P* = .001) in women using copper intrauterine devices. Although the frequency and density of beneficial lactobacilli did not change among intrauterine device users over 6 months, there was an increase in the log concentration of *G vaginalis* (4.7, 5.2, 5.8, 5.9; *P* = .046) and *A vaginae* (3.0, 3.8, 4.6, 5.1; *P* = .002) between baseline and 30, 90, and 180 days after initiation. Among other contraceptive groups, women using depot medroxyprogesterone acetate had decreased *L iners* (mean decrease log concentration = 0.8; 95% confidence interval, 0.3–1.5; *P* = .004) and there were no significant changes in beneficial *Lactobacillus* species over 180 days regardless of contraceptive method used.

**Conclusion:**

Copper intrauterine device use may increase colonization by bacterial vaginosis–associated microbiota, resulting in increased prevalence of bacterial vaginosis. Use of most hormonal contraception does not alter vaginal microbiota.

## Introduction

Reproductive-aged women commonly use and frequently change contraceptive methods. Understanding the impact of contraceptive initiation and use on vaginal microbiota is important since perturbations often cause distressing symptoms and bacterial vaginosis (BV) has been associated with increased risk of sexually transmitted infections,^1^, ^2^, ^3^ including HIV.^4^, ^5^, ^6^ Systematic review of studies evaluating the risk of HIV acquisition and contraceptive use suggests that depot medroxyprogesterone acetate (DMPA) may increase the risk of HIV acquisition.^7^ The possibility that contraceptive use may alter HIV susceptibility warrants further investigation of potential mechanisms, including understanding the impact on the vaginal microbiota.^8^

**AJOG at a Glance**

**Why was this study conducted?**

This study was conducted to evaluate the impact of contraceptive use on the vaginal microbiome of Zimbabwean women.

**Key findings**

Key findings include that hormonal contraceptive use did not alter vaginal microbiota over 6 months, while copper intrauterine device use was associated with increased bacterial vaginosis and associated microbiota, including *Gardnerella vaginalis* and *Atopobium vaginae*.

**What does this add to what is known?**

These data from a population of African women contribute to the body of evidence from the United States suggesting women using copper intrauterine devices are more likely to have changes in the vaginal microbiome including an increase in asymptomatic and symptomatic bacterial vaginosis.

BV is associated with shifts in vaginal microbiota, characterized by a change in dominant bacterial species from *Lactobacillus*-predominant to a mixture of anaerobic species.^9^, ^10^, ^11^ Women having a normal healthy pregnancy have lactobacilli as predominant members of the vaginal microbiome,^12^ and nonpregnant women having *Lactobacillus*-dominant microbiota have reduced susceptibility to HIV and other sexually transmitted infections.^13^, ^14^ BV as assessed by Nugent criteria is common in reproductive-aged women, with an overall prevalence of 29% in healthy US women.^15^ The impact of contraceptives on the vaginal microbiota and BV has been evaluated in several cross-sectional and longitudinal studies with inconsistent results. In these studies, women using oral contraceptives have generally been shown to have decreased risk of BV,^16^ while women using intrauterine devices (IUDs) have had inconsistent associations with prevalent BV.^17^, ^18^ In cross-sectional studies that include evaluation of the vaginal microbiome, women using DMPA or oral contraceptives were reportedly less likely to be colonized by BV-associated microbiota, while women using levonorgestrel (LNG)-releasing intrauterine systems (IUS) had a trend toward more BV-associated microbiota.^19^ There are fewer published longitudinal studies assessing the impact of contraceptives on vaginal microbiota, but those that have been published suggest that women using copper IUDs may have a modest increased risk of BV^20^ and women using the LNG-IUS had no increased risk of BV^21^ and no changes in the microbiome consistent with BV.^22^, ^23^

Our objective was to evaluate changes in prevalent BV and selected vaginal microbiota after initiation and use over 6 months of 6 contraceptive methods, including 3 hormonal injectables, 2 hormonal implants, and the copper IUD. We hypothesized that women initiating and using copper IUD would have increased BV and BV-associated microbiota compared to women initiating and using hormonal contraceptive methods.

## Materials and Methods

### Study population and sample collection

We performed a parallel longitudinal cohort study (ClinicalTrials.gov no: NCT02038335) of women initiating contraception with injectable (DMPA, norethisterone enanthate [Net-En], or medroxyprogesterone acetate and ethinyl estradiol [MPA/EE], implant (LNG or etonogestrel [ENG] subdermal implant), or intrauterine (copper T380A IUD [Cu-IUD] contraceptives. The primary objective was to assess the impact of initiation and continued use of contraceptives on HIV target cells in the lower genital tract at 1, 3, and 6 months of use and here we report on the secondary objective to assess the impact of contraceptive initiation and use on vaginal microbiota. The study was designed to assess changes compared to baseline with each woman serving as her own control; therefore, being free of exogenous steroid hormones at baseline and in a uniform phase of menses was central to the study design. Given the critical importance of the baseline values, laboratory confirmation by ultra-high-performance liquid chromatography tandem mass spectrometry (UPLC/MS/MS) was performed to evaluate serum progesterone, LNG, ENG, norethindrone, and MPA concentrations, which covered the full spectrum of regionally available contraceptive progestins at the time this study was conducted. Baseline sampling was performed at the enrollment visit when all enrolled women were free of hormonal or intrauterine contraceptive use for the preceding 30 days and free of DMPA use for the preceding 10 months by self-report. All samples from participants found to have exogenous synthetic progestin blood levels contradictory to self-reported nonuse were retested to confirm biological results and to rule out contamination during sample processing. All retesting confirmed original results and the participants were disqualified from the study.

We calculated a sample size of 37 women in each group would be needed to have 80% power to detect a 1-log change in microbial densities, based on a paired samples *t* test and a common SD of the microbial density difference of 2.1 observed in a prior study.^24^ To account for loss to follow-up, we planned a sample size of 40 women per contraceptive group.

The University of Pittsburgh Institutional Review Board and the Medical Research Council of Zimbabwe approved this study. All participants were enrolled at Spilhaus Family Planning Center in Harare, Zimbabwe, and signed informed consent before study participation.

The study population consisted of 451 women, age 18-34 years, seeking contraception in Harare, Zimbabwe. Eligible women were healthy, HIV negative, nonpregnant, and had regular menstrual cycles. Women were excluded if within 30 days of enrollment they: (1) used any hormonal or intrauterine contraceptive; (2) underwent any genital tract procedure (including biopsy); (3) were diagnosed with any urogenital tract infection; or (4) used any oral or vaginal antibiotics, oral or vaginal steroids, or any vaginal product or device except tampons and condoms (eg, spermicide, microbicide, douche, sex toys, and diaphragms). Women were also excluded if by self-report they used DMPA within 10 months of enrollment, were pregnant or breast-feeding within 60 days of enrollment, or had a new sexual partner within 90 days of enrollment. Exclusion criteria included having a contraindication, allergy, or intolerance to use of the contraceptive desired by the participant and having a prior hysterectomy or malignancy of the cervix or uterus.

Screening included urine pregnancy testing, 2 rapid HIV screening tests to rule out HIV infection, and collection of genital tract swabs for detection of *Neisseria gonorrhoeae*, *Chlamydia trachomatis* (ProbeTec; Becton Dickenson, Sparks, MD, or GeneXpert; Cepheid, Sunnyvale, CA), and *Trichomonas vaginalis* (OSOM; Sekisui Diagnostics, Lexington, MA).

Eligible participants presented for enrollment on a day when no vaginal bleeding was present and when they were in the follicular phase of menses (day 1-14) by self-reported last menstrual period. Participants were asked to refrain from any vaginal or anal intercourse for 48 hours prior to sample collection at enrollment and all follow-up visits. Participants selected their contraceptive group from among the 6 options (DMPA, Net-En, MPA/EE, LNG subdermal implant, ENG subdermal implant, and Cu-IUD) and a study clinician administered the selected contraceptive at the enrollment visit immediately following collection of all study samples. IUDs and implants were inserted per standard clinical practice. Participants were asked about reproductive tract symptoms at each follow-up visit and, if present, an adverse event was recorded and the symptom was diagnosed and treated as needed. All laboratory personnel were masked to clinical status of participants including contraceptive group.

### Laboratory methods

Vaginal swabs (polyester-tipped) for evaluation of BV using Nugent criteria^25^ and swabs for quantitative polymerase chain reaction (qPCR) analyses of vaginal microbiota (nylon-flocked swabs) (Puritan, Guilford, ME) were collected at enrollment, and 30, 90, and 180 days after initiation of contraceptive method. Vaginal swabs collected for Nugent analyses were immediately rolled onto glass microscope slides and air-dried and swabs collected for qPCR were immediately placed in a cryotube on ice. Nugent scores of 0-3 were considered normal (*Lactobacillus* dominant), scores of 4-6 were labeled as intermediate (mixed bacterial morphotypes), and scores of 7-10 were indicative of BV (absence of lactobacilli and predominance of other bacterial morphotypes). Five *Lactobacillus* species were included, including 3 species that have been linked with better reproductive health outcomes (*L crispatus*, *L gasseri*, *L jensenii*) as well as 1 species that is very prevalent and is not considered to be beneficial (*L iners*).^13^, ^14^ *Gardnerella vaginalis* and *Atopobium vaginae* were chosen as targets for this analysis because, in women with BV, they are the 2 most dominant members of the vaginal microbiota, and *Megasphaera* phylotype I was included because this novel species is specific to women with BV and is associated with genital inflammation.^26^

Blood samples were collected at each visit and analyzed for hormonal status as previously described.^27^ All samples were transported to the University of Zimbabwe-University of California, San Francisco Central Laboratory within 90 minutes of collection. Swabs for qPCR and blood aliquots were immediately stored at –80°C and shipped on dry ice to Magee-Womens Research Institute, Pittsburgh, PA, where testing was performed.

Bacterial DNA from vaginal swabs for qPCR was extracted using the QIAamp DNA mini kit (Qiagen, Valencia, CA) according to the manufacturers guidelines with modifications based on the observations by Yuan et al^28^ to maximize bacterial yield and diversity (method 1). Sham swabs (swabs without contact to human or bacterial DNA) were also subjected to extraction and processed in parallel with vaginal swab samples for extraction control. Species-specific primer sets previously developed were utilized for qPCR assays.^29^, ^30^ Specificity of the primer sets were evaluated for cross-reactivity by testing each set in the presence of pure DNA extracted using the QiaAMP DNA mini kit (Qiagen) from each of the 8 target organisms as described above. The *Lactobacillus* primer sets were also tested in the presence of DNA extracted from the following American Type Culture Collection (ATCC) (Manassas, VA) isolates and 1 well-characterized stock isolate: *L gasseri* (ATCC 9857), *L coleohominis* (stock isolate), *L fermentum* (ATCC 23271), *L jensenii* (ATCC 25258), *L johnsonii* (ATCC 33200), *L rhamnosus* (ATCC 21052), *L vaginalis* (ATCC 49540), and *L oris* (ATCC 49062). These lactobacilli were chosen because of their frequency of colonization in the vagina.^31^, ^32^ With the exception of the *L gasseri* primer and its cross-reactivity with *L johnsonii*, no cross-reactivity was observed with any of the other primer sets. No-template controls, consisting of all polymerase chain reaction (PCR) reagents with the exception of template DNA, were run to assess for well-to-well contamination. Endogenous positive controls (pure extracted DNA of the targeted species) were run to assess PCR inhibition and determination of specific melt curve temperatures for data analysis. Standard curves for absolute quantification constructed from *Escherichia* clones were prepared by diluting purified linearized plasmids containing our target of interest from 10^2^-10^9^ gene copies.

Vaginal swab samples, sham swabs, standards, endogenous positive controls, and no-template controls were run in triplicate and were detected and reported using a SYBR Green technology platform (Bio-Rad, Hercules, CA). Assays were performed on a CFX Connect real-time detection system (Bio-Rad). Each 20-μL reaction aliquoted into the well of a Hard Shell PCR plate (Bio-Rad) contained 1X SsoAdvanced universal SYBR Green supermix (Bio-Rad), 0.5 μmol/L of final concentration of forward and reverse primers, and 2 μL of template DNA. Plates were sealed with Microseal “B” adhesive optically clear seals (Bio-Rad). Temperature and cycling conditions for qPCR included an initial denaturation step of 98°C for 2 minutes, followed by 40 cycles of denaturation at 98°C for 30 seconds, and annealing at 62-65°C (primer dependent) for 30 seconds with fluorescence measured immediately following each cycle. Melt curve analysis to monitor nonspecific amplification or binding of dye was performed following initial amplification by heating from 65-95°C at 0.5°C increments, each held for 5 seconds. The formation of nontarget products or nonspecific binding was not observed. All melt peaks were within 1°C of the determined target temperature for organism-specific, pure DNA. All quantification cycles for no-template controls and sham swabs were >3.3 cycles above the quantification cycle for the lowest standard dilution (10^2^). The range of slopes observed for qPCR assays was –3.47 to –4.0, with an efficiency range of 77-94.1% and a linearity value of >0.99 for all assays. Our limit of detection for qPCR assays in this study ranged from 10^3^ or 10^4^-10^9^ gene copies per swab depending on the organism tested. The averages of bacterial concentrations were calculated and bacterial quantities were expressed as gene copies per swab.

### Statistical analysis

This analysis included 1047 visits for 266 women who were free of nonstudy exogenous hormones (confirmed by UPLC/MS/MS) throughout the study and who completed at least 90 days of follow-up. Descriptive statistics including frequencies, medians with interquartile range (IQR), and means with SD were used to characterize demographic and behavioral characteristics and differences between the contraceptive arms were assessed using Fisher exact, Pearson χ^2^, 1-way analysis of variance, and Kruskal-Wallis tests. Modified Poisson regression models^33^ were used to compare the marginal prevalence of vaginal microbiota and mixed effects generalized linear models with a random effect specified at the participant level^34^ were used to compare the mean difference in log quantity of vaginal microbiota (expressed as gene copies/swab) prior to and during contraceptive use. The log rank test was used to compare the incidence of symptomatic BV between Cu-IUD users and those using hormonal methods. All statistical tests were evaluated at the 2-side .05 significance level.

## Results

### Demographic characteristics

**Figure 1:**
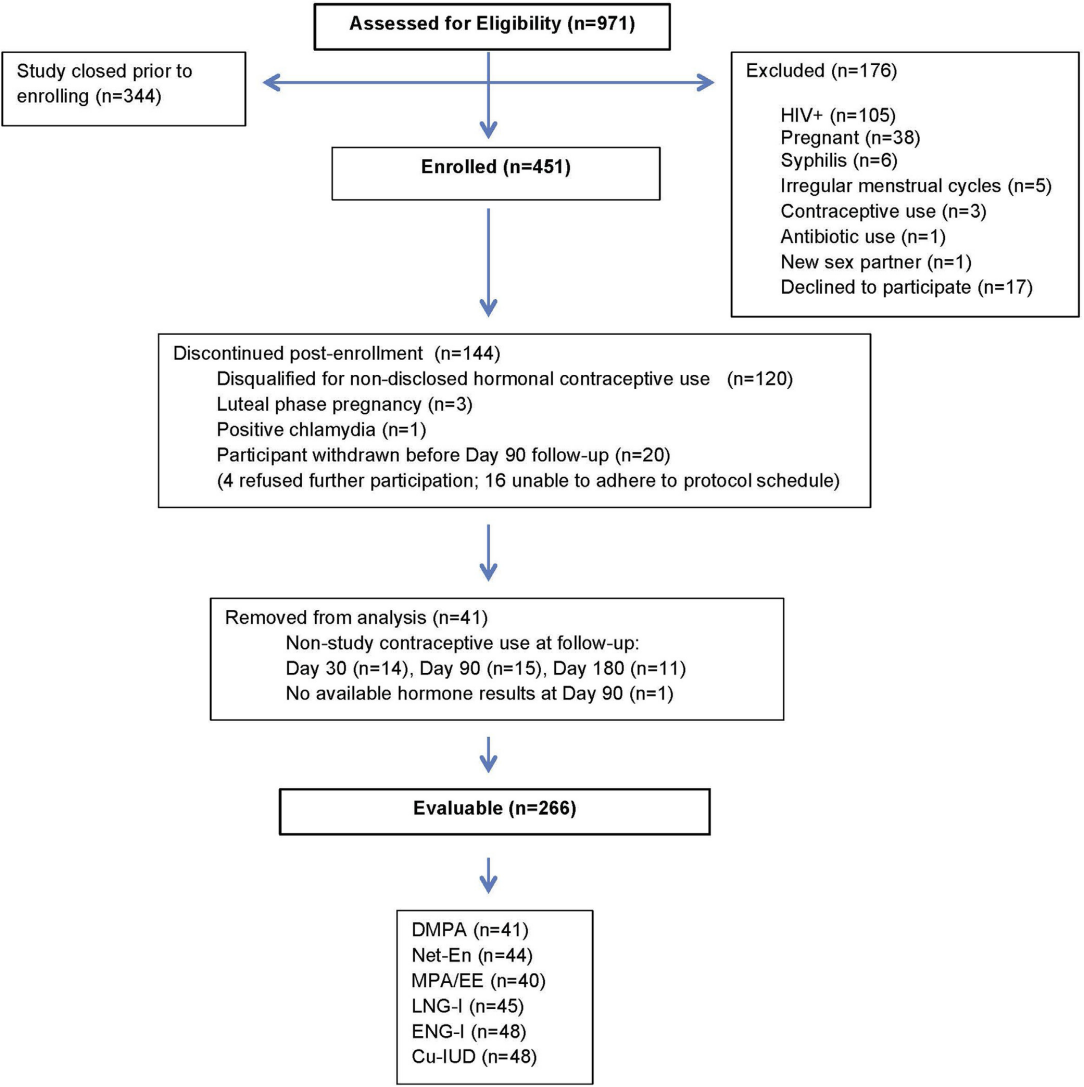
Diagram of participant flow from eligibilty assessment to final categorization Diagram of participant flow from eligibility assessment to final categorization. *Cu-IUD*, copper T380A intrauterine device; *DMPA*, depot medroxyprogesterone acetate; *EE*, ethinyl estradiol; *ENG-I*, etonogestrel subdermal implant; *LNG-I*, levonorgestrel subdermal implant; *MPA*, medroxyprogesterone acetate; *Net-En*, norethisterone enanthate. *Achilles et al. Impact of contraception on vaginal microbiota. Am J Obstet Gynecol 2018.*

From February 2014 through December 2015, 971 participants were assessed for study eligibility and 451 were enrolled. Of the 451 enrolled participants, 266 (59%) were evaluable. A flow diagram of all screened and enrolled participants is shown in Figure 1, including 124 (27%) disqualified for ineligibility after enrollment, 20 (4%) who did not complete follow-up through day 90, and 40 (9%) who had evidence of nonstudy hormone use at follow-up. Based on UPLC/MS/MS, 327 (73%) enrolled women were free of exogenous progestins at enrollment and of these 276 (84%) were in the follicular phase of menses (progesterone <1000 pg/mL) and 51 (16%) were not (progesterone ≥1000 pg/mL), with median progesterone values of 41 (IQR 12.5-64.8) and 4758 (IQR 2430-8245) pg/mL, respectively. Phase of the menstrual cycle (follicular vs nonfollicular) did not significantly impact BV prevalence by Nugent score or microbe quantity by qPCR (*P* > .09).

Evaluable participants were less likely to select DMPA (15% vs 26%) and more likely to select ENG subdermal implant (18% vs 9%) and Cu-IUD (18% vs 11%) compared to nonevaluable participants (*P* = .002). Evaluable participants were also older by 1 year (27 ± 4 years vs 26 ± 4 years, *P* = .01) and otherwise did not differ on any demographic or sexual behavioral feature compared to nonevaluable participants.

Among evaluable participants, women in self-selected contraceptive groups differed with respect to BMI, partner status, education, and frequency of intercourse, with women selecting DMPA to be leaner (*P* = .001) and women selecting copper IUD to be less likely living with a partner (*P* = .01), more likely to have achieved tertiary education (*P* = .02), and less frequently engaging in intercourse (*P* = .001) (Table 1). The proportion of participants with sexual partners did not differ between groups and only 6 of 266 (2%) participants in this study across all groups did not have a partner. Frequency of sexual intercourse was similar between the groups over the course of the study with the exception of increased sexual frequency at the 180-day follow-up visit among injectable contraceptive users compared to women using implants or Cu-IUD (*P* = .03).

**Table 1:** Demographic characteristics

Evaluable participants							
	DMPA n = 41	Net-En n = 44	MPA/EE n = 40	LNG-I n = 45	ENG-I n = 48	Cu-IUD n = 48	*P* value^a^
Age, y	26.6 ± 4.3	26.5 ± 3.8	28.3 ± 4.3	26.7 ± 4.0	27.4 ± 3.7	27.6 ± 4.3	.26^b^
Gravidity, median (IQR)	2 (1.5–3)	2 (1–2)	2 (2–3)	2 (1–3)	2 (1–3)		.17^c^
Parity, median (IQR)	2 (1.5–3)	2 (1–2)	2 (2–3)	2 (1–2.5)	2 (1–3)		.08^c^
Body mass index, kg/m^2^	23.2 ± 3.5	24.6 ± 4.4	27.1 ± 5.7	26.3 ± 4.2	25.3 ± 4.5	26.6 ± 5.1	.001^b^
Ethnicity							.96
Shona	39 (95.1%)	42 (95.5%)	38 (95.0%)	41 (91.1%)	45 (93.8%)	44 (91.7%)	
Ndebele	1 (2.4%)	2 (4.5%)	1 (2.5%)	1 (2.2%)	1 (2.1%)	1 (2.1%)	
Malawian	1 (2.4%)	0	1 (2.5%)	3 (6.7%)	2 (4.2%)	2 (4.2%)	
Zambian	0	0	0	0	0	1 (2.1%)	
Marital status							.15^d^
Single, never married	1 (2.4%)	1 (2.3%)	1 (2.5%)	1 (2.2%)	1 (2.1%)	5 (10.4%)	
Married	35 (85.4%)	39 (88.6%)	38 (95.0%)	41 (91.1%)	41 (85.4%)	32 (66.7%)	
Divorced	4 (9.8%)	2 (4.5%)	0	1 (2.2%)	4 (8.3%)	7 (14.6%)	
Separated	1 (2.4%)	2 (4.5%)	1 (2.5%)	1 (2.2%)	1 (2.1%)	4 (8.3%)	
Widowed	0	0	0	1 (2.2%)	1 (2.1%)	0	
Partner status							.01^d^
Lives with partner	35 (85.4%)	39 (88.6%)	37 (92.5%)	40 (88.9%)	42 (87.5%)	31 (64.6%)	
Does not live with partner	4 (9.8%)	5 (11.4%)	3 (7.5%)	5 (11.1%)	4 (8.3%)	15 (31.3%)	
Not applicable/none	2 (4.9%)	0	0	0	2 (4.2%)	2 (4.2%)	
Religious identification							.48
Christian	37 (90.2%)	42 (95.5%)	37 (92.5%)	43 (95.6%)	47 (97.9%)	45 (93.8%)	
Muslim	1 (2.4%)	1 (2.3%)	1 (2.5%)	2 (4.4%)	0	0	
African traditional religion	0	0	1 (2.5%)	0	0	0	
None	3 (7.3%)	1 (2.3%)	1 (2.5%)	0	1 (2.1%)	3 (6.3%)	
Education							.02^d^
None	0	0	0	1 (2.2%)	0	0	
Primary	8 (19.5%)	1 (2.3%)	6 (15.0%)	2 (4.4%)	8 (16.7%)	6 (12.5%)	
Secondary	33 (80.5%)	42 (95.5%)	34 (85.0%)	41 (91.1%)	38 (79.2%)	36 (75.0%)	
Tertiary	0	1 (2.3%)	0	1 (2.2%)	2 (4.2%)	6 (12.5%)	
Frequency of condom use in last 10 sexual encounters							.12^d^
0	25 (61.0%)	36 (81.8%)	31 (77.5%)	34 (75.6%)	33 (68.8%)	30 (62.5%)	
1–9	11 (26.8%)	2 (4.5%)	7 (17.5%)	6 (13.3%)	11 (22.9%)	8 (16.7%)	
10	5 (12.2%)	6 (13.6%)	2 (5.0%)	5 (11.1%)	4 (8.3%)	10 (20.8%)	
Typical frequency of intercourse/mo	12.9 ± 6.9	16.3 ± 6.5	13.9 ± 6.5	13.8 ± 6.4	15.2 ± 8.3	10.2 ± 7.1	.001^b^
Sexually transmitted infections at screening							
*Chlamydia trachomatis*	5 (12.2%)	0	2 (5.0%)	4 (8.9%)	1 (2.1%)	4 (8.3%)	.11
*Neisseria gonorrhoeae*	0	1 (2.3%)	1 (2.5%)	3 (6.7%)	0	0	.27
*Trichomonas vaginalis*	4 (9.8%)	1 (2.3%)	5 (12.5%)	3 (6.7%)	0	3 (6.3%)	.10

Data presented as mean ± SD or n (%) unless otherwise noted.

*Cu-IUD*, copper T380A intrauterine device; *DMPA*, depot medroxyprogesterone acetate; *EE*, ethinyl estradiol; *ENG-I*, etonogestrel subdermal implant; *IQR*, interquartile range; *LNG-I*, levonorgestrel subdermal implant; *MPA*, medroxyprogesterone acetate; *Net-En*, norethisterone enanthate.

*Achilles et al. Impact of contraception on vaginal microbiota. Am J Obstet Gynecol 2018*.

a Fisher exact test

b 1-way Analysis of variance

c Kruskal-Wallis test

d Pearson χ^2^ test.

### Impact of contraceptive initiation and use on vaginal microbiota

**Figure 2:**
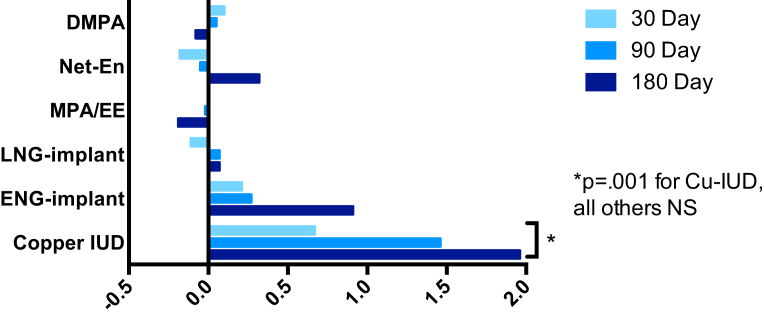
Change in Nugent score following contraceptive initiation and use Change in Nugent score from baseline at 30, 90, and 180 days following initiation and continuous use of contraceptive method. At enrollment following baseline sample collection, participant-selected study contraception was administered from available options including injectables (depot medroxyprogesterone acetate [DMPA], norethisterone enanthate [Net-En], or medroxyprogesterone acetate/ethinyl estradiol [MPA/EE]), subdermal implants (levonorgestrel [LNG] or etonogestrel [ENG]), or copper T380A intrauterine device [Cu-IUD]. All participants were confirmed by tandem mass spectrometry of plasma at each visit to be free of other exogenous hormones. *NS*, not significant at the .05 probability level. *Achilles et al. Impact of contraception on vaginal microbiota. Am J Obstet Gynecol 2018.*

Asymptomatic BV was detected at baseline by Nugent score in 83 (31%) of all evaluable participants. Asymptomatic BV prevalence increased in women initiating Cu-IUD from 27% at baseline, 35% at 30 days, 40% at 90 days, and 49% at 180 days (*P* = .005 compared to marginal prevalence at enrollment). Women initiating hormonal methods had no change in BV prevalence over 180 days (Table 2). The mean increase in Nugent score was 1.2 (95% confidence interval [CI], 0.5–2.0; *P* = .001) over all follow-up visits and 2.0 (95% CI, 0.9–3.0; *P* <.001) by 180 days in women using Cu-IUD. There was no change in Nugent score in women initiating hormonal methods over 180 days (Figure 2). Based on clinical diagnosis, the incidence of symptomatic BV requiring treatment was 17.5 per 100 woman years in the Cu-IUD group and 2.9 per 100 woman years in the hormonal contraceptive groups combined (*P* = .007). No participants were diagnosed with pelvic inflammatory disease.

**Table 2:** Prevalence of bacterial vaginosis over time following contraceptive initiation

	Enroll	30 d	90 d	180 d	*P* value^a^
DMPA	29.3%	30.0%	31.7%	30.8%	.77
Net-En	40.9%	38.6%	40.9%	46.3%	.34
MPA/EE	30.0%	35.0%	35.0%	38.9%	.21
LNG implant	35.6%	35.6%	42.2%	39.5%	.27
ENG implant	25.0%	22.9%	25.0%	36.2%	.97
Copper IUD	27.1%	35.4%	39.6%	48.9%	.005

Data presented as proportion of women with Nugent score ≥7 at each visit.

*DMPA*, depot medroxyprogesterone acetate; *EE*, ethinyl estradiol; *ENG*, etonogestrel (subdermal); *IUD*, intrauterine device; *LNG*, levonorgestrel (subdermal); *MPA*, medroxyprogesterone acetate; *Net-En*, norethisterone enanthate.

*Achilles et al. Impact of contraception on vaginal microbiota. Am J Obstet Gynecol 2018*.

a From modified Poisson regression model comparing marginal prevalence at follow-up visits to marginal prevalence at enrollment.

**Figure 3:**
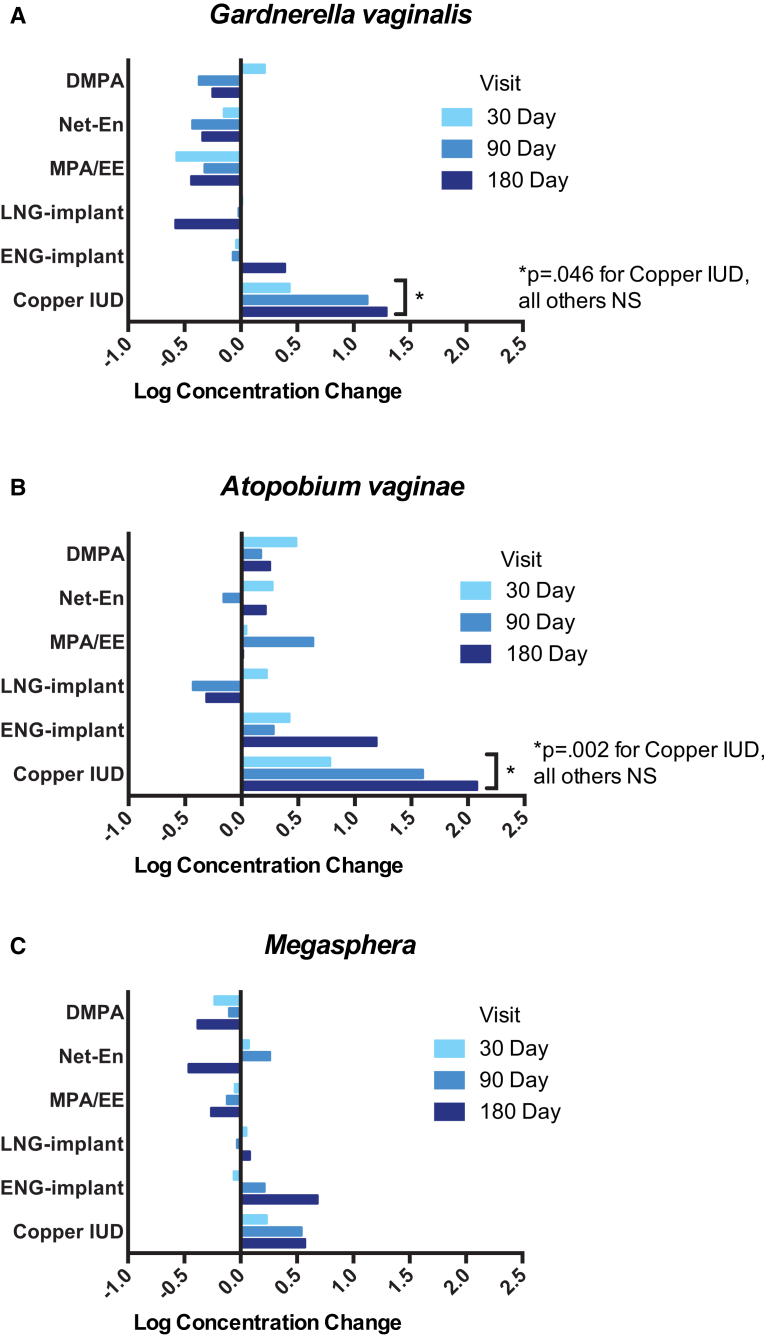
Changes in bacterial vaginosis-associated microbiota following contraceptive initiation and use Change in log concentration of vaginal **A**, *Gardnerella vaginalis*, **B**, *Atopobium vaginae*, and **C**, *Megasphaera*-like bacterium phylotype I from baseline at 30, 90, and 180 days following initiation and continuous use of contraceptive method. At enrollment following baseline sample collection, participant-selected study contraception was administered from available options including injectables (depot medroxyprogesterone acetate [DMPA], norethisterone enanthate [Net-En], or medroxyprogesterone acetate/ethinyl estradiol [MPA/EE]), subdermal implants (levonorgestrel [LNG] or etonogestrel [ENG]), or copper T380A intrauterine device [IUD]. All participants were confirmed by tandem mass spectrometry of plasma at each visit to be free of other exogenous hormones. *NS*, not significant at the .05 probability level. *Achilles et al. Impact of contraception on vaginal microbiota. Am J Obstet Gynecol 2018.*

**Figure 4:**
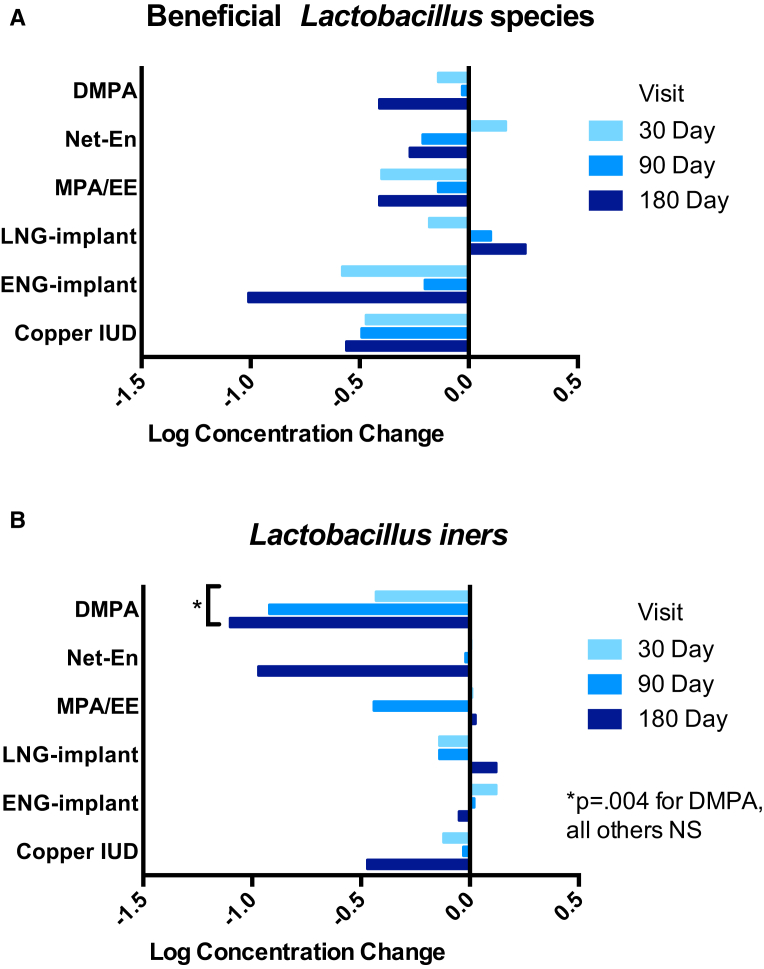
Changes in *Lactobacillus* species following contraceptive initiation and use Change in log concentration of vaginal **A**, beneficial *Lactobacillus* species, including *L crispatus*, *L jensenii*, and *L gasseri*/*johnsonii* group and **B**, *L iners* from baseline at 30, 90, and 180 days following initiation and continuous use of contraceptive method. At enrollment following baseline sample collection, participant-selected study contraception was administered from available options including injectables (depot medroxyprogesterone acetate [DMPA], norethisterone enanthate [Net-En], or medroxyprogesterone acetate/ethinyl estradiol [MPA/EE]), subdermal implants (levonorgestrel [LNG] or etonogestrel [ENG]), or copper T380A intrauterine device [IUD]. All participants were confirmed by tandem mass spectrometry of plasma at each visit to be free of other exogenous hormones. *NS*, not significant at the .05 probability level. *Achilles et al. Impact of contraception on vaginal microbiota. Am J Obstet Gynecol 2018.*

There was an increase in the log concentration of *G vaginalis* (4.7, 5.2, 5.8, 5.9) and *A vaginae* (3.0, 3.8, 4.6, 5.1) between baseline and 30, 90, and 180 days after initiation of Cu-IUD. The mean change over all follow-up visits in *G vaginalis* and *A vaginae* in Cu-IUD users was 0.7 (95% CI, 0.01–1.4; *P* = .046) and 1.3 (95% CI, 0.5–2.2; *P* = .002), respectively (Figure 3, A and B). Among hormonal contraceptive groups, there were no significant changes in BV-associated microbiota (Figure 3). Although the frequency and density of beneficial lactobacilli (including *L crispatus*, *L jensenii*, and *L gasseri*/*johnsonii* group) did not significantly change among Cu-IUD or hormonal contraceptive users over the 4 visits (Fig 4, A), women using DMPA had a decreased concentration of *L iners* (mean decrease in log concentration was 0.8; 95% CI, 0.3–1.5; *P* = .004) over 180 days (Fig 4, B).

## Comment

In this study, we found increased colonization by the BV-associated microbiota *G vaginalis* and *A vaginae*, as well as increased prevalence of BV during the 6-month study duration in women who opted to initiate and use copper IUD. Since women in this study who chose IUDs reported a somewhat lower frequency of vaginal intercourse relative to women who chose one of the other contraceptives, the positive association between BV and IUD cannot be explained by increased sexual activity. Use of hormonal contraceptives over 6 months did not appear to significantly shift vaginal microbial populations including beneficial *Lactobacillus* species or common BV-associated species.

To independently and specifically evaluate the impact of contraceptives, we measured hormonal status at every visit using UPLC/MS/MS and women served as their own controls allowing evaluation of longitudinal changes from baseline, constituting strengths of this study. Further, we used qPCR to assess changes in quantities of common beneficial and BV-associated microbiota. Published studies reporting the impact of contraceptives on vaginal microbiota have limitations including participant self-report of contraceptive method^27^, ^35^, ^36^ and use of heterogeneous comparison groups, wherein IUD users and those who have had tubal ligations have been combined,^20^ or women using the LNG-IUS have been compared to women using a variety of short-acting hormonal contraceptive methods, including oral contraceptives, contraceptive vaginal rings, and contraceptive patches.^21^ This approach has made it difficult to interpret some of the studies since each contraceptive method has not been evaluated independently in a prospective manner.

Our study has limited ability to address the common practices of frequent contraceptive switching and simultaneous use of multiple contraceptives. Further, since this is a prospective cohort study and participants self-selected contraceptive methods there may be differences, including behavioral differences, among women who chose IUDs compared to those who chose a hormonal method. The majority of hormonal contraceptive methods studied here were of progestin-only methods and only 1 arm consisted of a combined hormonal method (MPA/EE) limiting power for analyzing combined hormonal methods separately. Given that estrogen induces the accumulation of glycogen in the vaginal epithelium and glycogen positively influences colonization by lactobacilli, it is reasonable to hypothesize that synthetic estrogen-containing methods may confer protection from BV. Notably, the LNG-IUS, which is more commonly used by US women compared to the Cu-IUD, was not included in this study. The LNG-IUS will be of interest for further study as it is both intrauterine and hormonal and therefore, unclear if it is likely to impact the vaginal microbiota.

If DMPA use is found to increase HIV acquisition risk, it is unlikely to do so by the mechanism of alteration of vaginal microbiota. Currently, there are insufficient available data to assess if there is an impact of copper IUD use on risk of HIV acquisition, largely because use of copper IUDs among women in high HIV prevalence areas has historically been low. However, the copper IUD is included as a comparator arm in the ongoing ECHO trial (NCT02550067), designed to assess HIV acquisition risk in women randomized to DMPA, LNG implant, and copper IUD and if copper IUD use is found to have an association, then alteration of vaginal microbiota may be a contributing factor. Clinically, women with recurrent BV may prefer to opt for a hormonal method rather than a copper IUD for contraception to minimize recurrence risk.
