# June 2018 (vol. 218, no. 6, pages 622.e1-10)

**DOI:** 10.1016/j.ajog.2021.01.034

**Published:** 2021-10

Achilles SL, Austin MN, Meyn LA, et al. Impact of contraceptive initiation on vaginal microbiota. Am J Obstet Gynecol 2018;218:622.e1-10.

In an evaluation of the impact of 6 forms of contraception on the vaginal microbiome of women in Zimbabwe, the abstract stated that subjects in the injectable contraception group had received 1 of the following: depot medroxyprogesterone acetate, norethisterone enanthate, or medroxyprogesterone acetate and ethinyl estradiol.

The third-named (combined) method consisted of medroxyprogesterone acetate plus estradiol cypionate, not ethinyl estradiol. This error appeared, either spelled out or with the incorrect abbreviation (MPA/EE rather than MPA/EC), in the summary on the opening page of the article (page 622.e1, under Study Design) and was repeated throughout the text (Materials and Methods, page 622.e2, column 1, paragraph 1, line 7; column 1, line 4, page 622.e3; column 2, line 15, page 622.e9), figures (Figure 1, page 622.e3, final block of flowchart and key; Figure 2, page 622.e6, third category in graph and key; Figure 3, page 622.e7, third category in sections A, B, and C of graph and key; Figure 4, page 622.e8, third category in sections A and B and key), and tables (Table 1, p. 622.e5, heading 3 under “Evaluable participants” and key; Table 2, page 622.e5, column 1, row 3, and key).

